# White Matter Language Pathways and Language Performance in Healthy Adults Across Ages

**DOI:** 10.3389/fnins.2019.01185

**Published:** 2019-11-01

**Authors:** James Houston, Jane Allendorfer, Rodolph Nenert, Adam M. Goodman, Jerzy P. Szaflarski

**Affiliations:** ^1^Department of Neurology, UAB Epilepsy Center, The University of Alabama at Birmingham, Birmingham, AL, United States; ^2^Departments of Neurosurgery and Neurobiology, The University of Alabama at Birmingham, Birmingham, AL, United States

**Keywords:** diffusion MRI, fractional anisotropy, tract based spatial statistics, white matter language pathways, inferior fronto-occipital fasciculus

## Abstract

The goal of this study was to determine the relationship between age-related white matter changes, with a specific focus on previously identified language pathways, and language functioning in healthy aging. 228 healthy participants (126 female; 146 right-handed), ages 18 to 76, underwent 3.0 Tesla MR diffusion weighted imaging (DWI) and a battery of language assessments including the Boston Naming Test (BNT), the Peabody Picture Vocabulary Test (PPVT), the Controlled Oral Word Association Test (COWAT), Semantic Fluency Test (SFT), and a subset of the Boston Diagnostic Aphasia Examination (CI-BDAE). Using tract based spatial statistics (TBSS), we investigated measurements of fractional anisotropy (FA), axial diffusivity (AD), radial diffusivity (RD), and mean diffusivity (MD). TBSS was used to create a white matter skeleton that was then used to analyze white matter changes (indexed by FA, AD, RD, and MD) with age and language performance. Results focused primarily on significant relationships (*p* < 0.05, cluster-wise FDR corrected for multiple comparisons) in the canonical language white matter pathways. We found a diffuse linear *decrease* with age in global white matter FA and a significant focal *increase* in FA with age within the bilateral superior cerebellar peduncles (SCPs). We observed that increased BNT scores were associated with increased FA within the left SLF, and within the posterior and antero-lateral portions of the right inferior frontal-occipital fasciculus (IFOF). Increased SFT and PPVT scores were associated with increased FA within the posterior portion of the right IFOF and increased FA within the left body of the corpus callosum was associated with lower COWAT scores. We found no association between FA and BDAE. MD, RD, and AD, were found to be inversely proportional to FA within the IFOF, with AD showing a negative correlation with SFT, and RD and MD showing a negative correlation with BNT. There was no association between CI-BDAE and any of the white matter measures. Significant differences between sexes included more pronounced FA decrease with age within the right SLF in males vs. females; there were no differences in language performance scores between sexes. We also found that there was no decline in language testing scores with increasing age in our cohort. Taken together, our findings of varying relationships between DTI metrics and language function within multiple regions of the non-dominant IFOF suggest that more robust language networks with bilateral structural connectivity may contribute to better overall language functioning, regardless of age.

## Introduction

Age-related changes in brain volume have been investigated using a variety of MRI and post-processing techniques, including alterations in white and gray matter volume and integrity (Steen et al., 1995; Cho et al., 1997; Hanyu et al., 1997; Tang et al., 1997, 2001; Ketonen, 1998; Bartzokis et al., 2001; Abe et al., 2002; Ge et al., 2002a, b; Hugenschmidt et al., 2008). In addition to pure anatomical investigations showing age-related changes in healthy aging, studies of patients with cognitive dysfunction have documented progressive degradation of the white matter associated with age and the degree of cognitive dysfunction (Ylikoski et al., 1993; Inzitari, 2000; Inzitari et al., 2000; Takahashi et al., 2004; Shenkin et al., 2005; Miranda et al., 2008; Madden et al., 2009; Ziegler et al., 2010). There is, however, a relative paucity of data concerning white matter structure, aging, and the relationship between those variables and the level of language functioning (Madhavan et al., 2014). In contrast, rich pediatric literature explores the processes of brain maturation and language development in numerous cross-sectional and longitudinal studies using structural and functional neuroimaging (Holland et al., 2001, 2007; Schmithorst et al., 2002, 2005; Szaflarski et al., 2006, 2012). In general, this literature demonstrates significant correlations between white matter structure and IQ, age-related changes in white matter maturation, and increased left-hemispheric participation in language-related processing with age. However, it is not well understood whether the microstructural integrity of white matter pathways associated with language in healthy adults predicts changes in language function or lateralization. Thus, the primary aim of the current study was to assess the relationship between white matter changes, language function and lateralization, and age in healthy adults.

Concerning white matter and language function, several specific white matter tracts have been found to contribute to language function including the arcuate fasciculus (AF), uncinate fasciculus (UF), inferior longitudinal fasciculus (ILF), superior longitudinal fasciculus (SLF), and inferior frontal-occipital fasciculus (IFOF) (Middlebrooks et al., 2017). The SLF is further divided into three subsections. Similar to lesions of the Broca’s area, disruptions in the dominant SLF III are associated with dysarthria/anarthria and speech output deficits (Bizzi et al., 2012). Disruptions of the dominant IFOF pathway are typically associated with anomia and paraphasic errors (Martino et al., 2010). The ILF is a multilayer and bidirectional tract that is involved in orthographic processing and visual cognition; disruption of this tract may be associated with various neuropsychological abnormalities such as visual agnosia or hallucinations, and alexia (Zemmoura et al., 2015). The UF is involved in semantic processing, with some evidence to suggest that disruption of the pathway can result in problems with proper name anomia and famous face recognition (Papagno et al., 2016). The AF pathway is considered one of the most important language pathways. For example, disruption of the AF has been related to difficulties in comprehension and repetition when combined with cortical dysfunction (Breier et al., 2008; Allendorfer et al., 2016). The ventral WM tracts, including the IFOF and ILF, are considered association fibers that run through the length of the temporal lobe. The ILF connects the temporal pole, limbic network, and anterior portions of the middle and inferior temporal gyri to the occipital lobe (Herbet et al., 2018). The IFOF courses medially to the ILF in the temporal lobe and connects the inferior orbitofrontal cortex with the inferior and medial occipital lobe (Catani and Thiebaut de Schotten, 2008). The UF is thought to share cortical projections with the ILF and, therefore, may assist in tasks that require temporal to frontal connections such as word retrieval and aspects of naming (Lu et al., 2002; Duffau et al., 2014).

Of the varying MRI acquisition techniques used to study age-related white matter changes, diffusion weighted imaging (DWI) has proven to be a robust marker of white matter structural integrity (Salat et al., 2005a, b; Yoshiura et al., 2005; Zhang et al., 2005; Lehmbeck et al., 2006; Hsu et al., 2008, 2010; Thomas et al., 2008; Yoon et al., 2008; Davis et al., 2009; Giorgio et al., 2010). DTI analysis provides a quantitative measure of the integrity of white matter tracts assessed indirectly by measuring the directionality of water diffusion through fractional anisotropy (FA), and varying rates of water diffusion through mean diffusivity (MD), radial diffusivity (RD), and axial diffusivity (AD) (Pierpaoli and Basser, 1996; Madden et al., 2004). Multiple previous studies have shown global decreases in white matter FA with normal aging especially from the 4th decade on (Courchesne et al., 2000) with regional decreases noted in the internal capsule, corpus callosum, and frontal, parietal, and occipital regions (Abe et al., 2002; Moseley, 2002; Hsu et al., 2010; Lebel et al., 2012). The age-related white matter degradation has also been suggested to occur in an anterior to posterior gradient, with the prefrontal regions and anterior portions of the corpus callosum (genu) being most significantly affected (Sullivan and Pfefferbaum, 2006; Madden et al., 2009; Zahr et al., 2009).

Tract-based spatial statistics (TBSS), a methodological tool that extends the evaluation of DTI data by providing great anatomical specificity in an objective manner (Smith et al., 2006, 2007). Longitudinal assessment with TBSS has previously shown annual decrease in FA and increases in AD, RD, and MD within white matter regions for older adults suggesting that these indices together may track axonal injury and reduced pathway coherence across the lifespan (Sexton et al., 2014). Although prior literature suggests that cognitive decline is partially related to global degradation of white matter over time, there is paucity of data investigating language performance in correlation with changes in white matter pathways associated specifically with language (Gerstenecker et al., 2017; Rabin et al., 2018). Given that some individuals develop language dysfunction with age, with or without the development of global cognitive impairment, it is important to understand whether age-related changes in white matter pathways contribute to the decline in specific language domains. Thus, direct comparison of measures of white matter integrity using TBSS with linguistic measures may help further outline these relationships.

Concerning the investigations of the interactions between language and DTI, few studies have examined the relationship between white matter FA values, anatomy, aging, and specific language function(s). *Decreased* FA in the left temporo-parietal white matter region has been correlated with poorer reading and also may have implications in patients with dyslexia (Klingberg et al., 2000). This region contains axons from AF and external capsule that project from the temporal, occipital, and inferior parietal cortices to the frontal cortex. In contrast, *increased* FA in the left perisylvian parietal and inferior frontal white matter is associated with faster lexical decision making (Gold et al., 2007). Improved grammar learning success (the ability to produce complex, rule-based speech) has been shown to have a positive correlation with increased FA in left hemispheric white matter tracts traveling posteriorly from Broca’s area via the SLF and anteriorly to the right prefrontal cortex (Floel et al., 2009). Another study in healthy elderly individuals showed that normal appearing white matter had reduced FA in ILF compared to younger adults which has implications in language function (Obler et al., 2010). A reduction in FA in the posterior portion of the SLF resulted in more difficulty in word retrieval in the elderly (Stamatakis et al., 2011). Lastly, reductions in FA in UF may play a role in slowing of lexical information processing in the aging brain (Kemmotsu et al., 2012).

The goal of this study was to determine the relationship between age-related white matter changes, with a specific focus on previously identified language pathways, and language functioning in healthy aging in a large sample of male and female participants across all degrees of handedness using DTI and whole brain TBSS. The secondary goal was to help to improve our understanding of the participation of various brain regions in language functioning including regions not included in the previously accepted classical language model (Geschwind and Galaburda, 1985). We hypothesized that degradation of global white matter pathways would show consistent age-related decline. We also hypothesized that a decrease in FA within the specific language-associate white matter pathways would correlate with poorer language functioning as measured with standard linguistic tests.

## Materials and Methods

### Participants

Data from two-hundred and forty-nine healthy subjects (158 females, 43 females; age range = 18–76) were analyzed for the present study (Allendorfer et al., 2016; Nenert et al., 2017; Nair et al., 2019). Eight of the enrolled participants were unable to complete language testing and/or imaging. Of the 241 participants who completed both DTI scanning and language assessments 13 were excluded upon visual inspection of the data because of excessive artifact (e.g., movement) leaving 228 participants to be included in the final analyses (Mean age = 40.85, SD = 14.68). There were 102 male and 126 female subjects, 81 with atypical handedness and 147 being right-handed. An emphasis was placed on oversampling participants with atypical handedness in order to provide sufficient representation of right handed vs. atypically handed subjects to address the analyses intended in the study. Subject recruitment was performed from December 2008 to January 2014 by word of mouth and institutional advertisements. This research was approved by the University of Alabama at Birmingham (UAB), Cincinnati Children’s Hospital Medical Center (CCHMC), and University of Cincinnati (UC) Institutional Review Boards. All study procedures, including language testing and MR imaging were performed in accordance with the ethics principles of the Declaration of Helsinki and the principles of informed consent. Each subject provided written informed consent prior to participation in the study. Each subject was confirmed to have no known contraindications to receiving MRI at 3.0 Tesla. We confirmed the health status of each participant through a battery of questions evaluating for any ongoing neurological or psychiatric conditions or the presence and/or absence of any pre-existing conditions.

### MRI Data Acquisition

Two hundred and one healthy subjects had imaging performed at the CCHMC Imaging Research Center (IRC) using an 8-channel head coil on a 3.0 T Philips MR system. An additional 48 subjects were scanned using a circular polarized head coil on a 3.0 T Siemens MR system at the University of Alabama at Birmingham’s Civitan Functional Neuroimaging Laboratory (CFNL). Initially, a three-plane localizer scan was performed followed by a shim procedure to generate a homogeneous magnetic field. Next, for localization of brain activation maps, anatomical scans were acquired. A high-resolution T1-weighted 3D anatomical scan was attained for brain localization utilizing a magnetization-prepared rapid acquisition with gradient echo (MP-RAGE) sequence. Parameters for the MP-RAGE performed in CCHMC IRC were as follows: TR/TE = 8.1/3.7 ms, flip angle = 8^°^, matrix 252 × 210, FOV 25.0 × 21.0 × 18.0 cm, slice thickness = 1 mm. At the CFNL, MP-RAGE parameters were as follows: TR/TE = 2300/2.17 ms, flip angle = 9^°^, matrix 256 × 256, FOV 25.6 × 25.6 × 19.2 cm, slice thickness = 1 mm. The DTI was performed using an echo planar image sequence and designed to keep the *b*-values and number of diffusion directions consistent between scanners. This was completed by using one image with no diffusion weighting (*b* = 0 s/mm^2^) and DWIs in 32 distinct directions (*b* = 800 s/mm^2^). This allowed us to obtain the same number of specific directions for tensor estimation, while also keeping in line with the 30 directions required for reliable estimation of the tensor orientation (Jones, 2004). At the IRC, the DTI parameters were as follows: TR/TE = 9403/69 ms, matrix 76 × 67, FOV 18.0 × 16.1 cm, slice thickness = 2.37 mm. The DTI parameters at the CFNL were TR/TE = 9400/89, matrix 96 × 96, FOV 24.0 × 24.0 cm, slice thickness = 2.5 mm.

### Language Assessments

All participants received a linguistic battery. The Boston Naming Test (BNT), Second Edition was utilized to evaluate naming performance (Kaplan et al., 2001). The Peabody Picture Vocabulary Test (PPVT), Fourth Edition assessed receptive vocabulary (Dunn and Dunn, 2007). Two tests of verbal fluency were scored: one based on the number of words generated in 1 min for a given letter [Controlled Oral Word Association Test (COWAT)] (Lezak, 1995) and one based on the number of words generated for a given category [Semantic Fluency Test (SFT)] (Cullum et al., 1995; Lezak, 1995). Finally, all participants received the Complex Ideation subset of the Boston Diagnostic Aphasia Examination (CI-BDAE) to test oral comprehension and recall of information (Goodglass and Kaplan, 1972).

### TBSS Data Processing

Diffusion weighted imaging data underwent standard pre-processing and diffusion tensor modeling to produce FA, MD, RD, and AD voxel-wise maps for each subject (Allendorfer et al., 2012). First, FSL was used to perform TBSS on each subject’s FA map using previously established procedures to skeletonize the data in preparation for statistical analyses (Smith et al., 2006; Allendorfer et al., 2012). This process included TBSS non-linear registration of FA data into the FMRIB58_FA standard-space image (1 × 1 × 1 mm). For each FA map, normalized images were then averaged to establish a group mean FA image. This was then used by the FA skeletonization program to create the group’s mean FA skeleton. The mean FA skeleton image is thought to reflect the center of fiber bundles. The FA skeleton image was thresholded at a value of 0.2 in order to suppress regions of partial gray matter and/or high inter-subject variability while also including the major white matter tracts. Finally, each subject’s FA data were projected onto the mean FA skeleton in order to create a 4-dimensional image file containing the skeletonized FA data for all subjects. This 4-dimensional FA skeleton image file was then used for statistical analyses.

For each of the other DTI metrics (MD, RD, AD), the TBSS process involved applying the same non-linear transformations used to register the FA data, combining all the subjects’ data into a 4-dimensional image file, then projecting these data onto the mean FA skeleton described above. This process resulted in a 4-dimensional skeleton image file for each of the other DTI metrics. These 4-dimensional skeleton image files for MD, RD and AD were then used for statistical analyses.

### Statistical Analysis of Skeletonized TBSS Data

Statistical analyses on the 4-dimensional skeleton image files for FA, MD, RD and AD were performed using SPM12 (statistical parametric mapping^1^). Regression analyses were computed using SPM12 between individual FA, MD, RD, and AD skeletonized maps and (1) subjects’ age (controlled for sex, handedness, and MRI scanner) and (2) language assessment performance (controlled for age, sex, handedness, and MRI scanner). Results were considered significant at *p* < 0.05, cluster-wise FDR corrected for multiple comparisons. A two sample *t*-test corrected for handedness compared white matter FA between sexes using age as an interaction variable. Location within specific white matter tracts for regions showing significant results were determined using the JHU ICBM-DTI-81 white matter atlas (Mori et al., 2005).

## Results

The final study population consisted of 228 participants with ages ranging from 18 to 76 (40 ± 14.7 years). There were 102 male (37 with atypical and 65 with right-handers), and 126 female subjects (44 with atypical and 82 with right-handers). Subjects were considered neurologically normal based on the interview and the lack of any underlying neurological diagnoses. In addition, Mini Mental State Examination (MMSE) (Folstein et al., 1975) performed on all participants showed a mean score of 29.3 (±1.28) indicating normal cognitive status.

### Behavioral Testing Results

For each behavioral score, a two-way ANOVA was computed with handedness and sex as factors (Table 1). No significant factor or interaction was found, indicating no differences between left-handed and right-handed participants and between sexes in language scores.

**TABLE 1:** Demographic and performance characteristics for subjects scanned at Cincinnati Children’s Hospital Medical Center (CCHMC) and University of Alabama at Birmingham (UAB).

**Demographic and assessment variable^a^**	**CCHMC**		**UAB**	
*N*, % females	184 (56.5%)		44 (52.2%)	
Age in years, mean (SD)	40.4 (15.1)		42.54 (12.3)	
Language assessment, mean (SD)				
Boston Naming Test score	56.6 (3.7)		53.8 (5.8)	
Peabody Picture Vocabulary Test	213.5 (9.3)		209 (18)^∗^	
Controlled Oral Word Association Test	40.1 (11.3)		39.1 (11.2)	
Semantic Fluency Test	56.9 (11.6)		56.4 (17.3)	
Boston Diagnostic Aphasia Examination	11.54 (0.85)		11.25 (1.1)	

**Language Assessment Variable**	**Mean (SD)**	**Hand**	**Sex**	**Hand × sex**

Boston Naming Test	56.1 (4.3)	1.23 (0.27)	1.89 (0.17)	0.51 (0.47)
Peabody Picture Vocabulary Test	212.6 (11.6)	2.15 (0.14)	1.87 (0.17)	0.58 (0.45)
Controlled Oral Word Association Test	39.9 (11.3)	0.12 (0.73)	1.2 (0.27)	0.28 (0.59)
Semantic Fluency Test	56.8 (12.8)	0.03 (0.85)	3.73 (0.06)	0.67 (0.41)
Boston Diagnostic Aphasia Examination	11.5 (0.9)	1.56 (0.21)	0 (0.99)	0.15 (0.69)

*For the combined sample, we present the mean and standard deviation for each behavioral score. In addition, two-way ANOVAs were computed, *F*-value and *p*-value are provided for each factor (handedness and sex) and for interaction between factors with *p*-values indicated in parentheses. ^*a*^Data are reported as mean (SD) for data except for number of subjects (*N*), which are reported as frequency (%). ^∗^A significant difference was found between sites for PPVT (two sample *t*-test, *p* = 0.02).*

### Language Testing and Age

**FIGURE 1:**
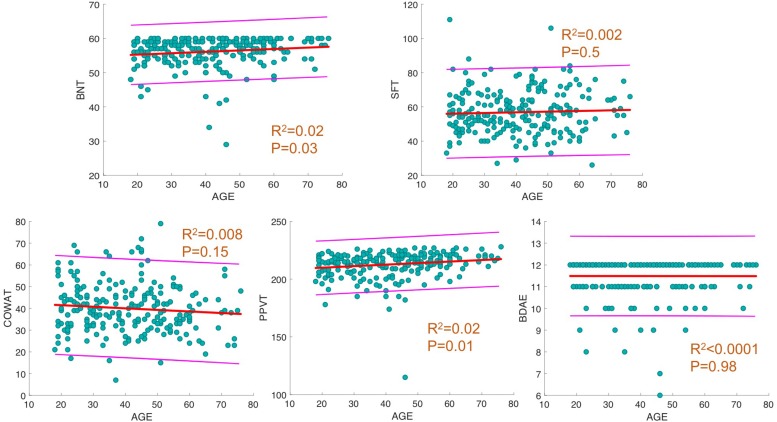
Linear fit between age and behavioral scores. For each behavioral score, linear fit of data with 95% prediction interval is depicted. In addition, *R*^2^ and *p*-values of regression analysis between age and score is provided.

For each behavioral score, linear fit of the data with a 95% prediction interval was determined, along with regression analysis between age and language scores. The results showed a significant increase in BNT and PPVT with progressing age (*p* = 0.02, *R*^2^ = 0.02; Figure 1) and no significant changes in the other measures.

### Language Testing and DTI Metrics

Regression analyses were performed on all subjects’ white matter skeletonized maps of each diffusion metric (FA, MD, RD, AD) and computed between language assessment performance (controlled for age, sex, handedness, and MRI scanner). All correlations discussed below were found to be significant at *p* < 0.05 corrected for multiple comparisons.

**FIGURE 2:**
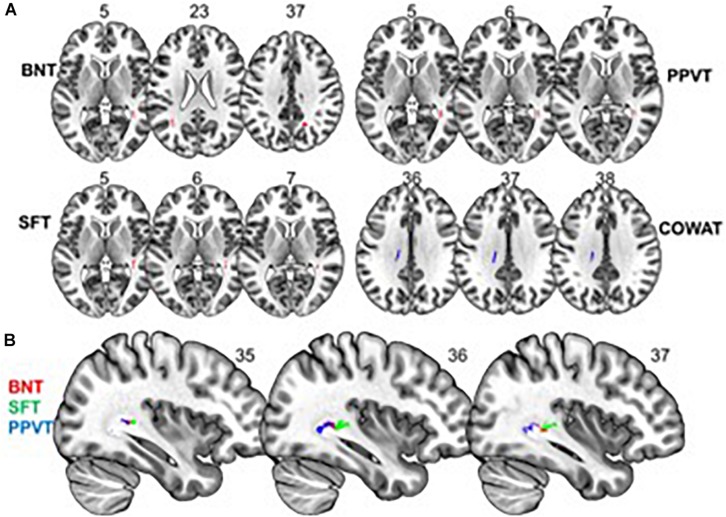
Significant correlations between FA and behavioral scores (*p* < 0.05, cluster-wise FDR corrected for multiple comparisons). **(A)** FA shows positive relationships with BNT, PPVT, and SFT performance and a negative relationship with COWAT performance. **(B)** Significant correlations between FA and BNT, SFT, and PPVT overlap in a region of the right inferior fronto-occipital fasciculus. Pictures are in neurological convention with left side in the figure corresponding to the left side of the brain.

**FIGURE 3:**
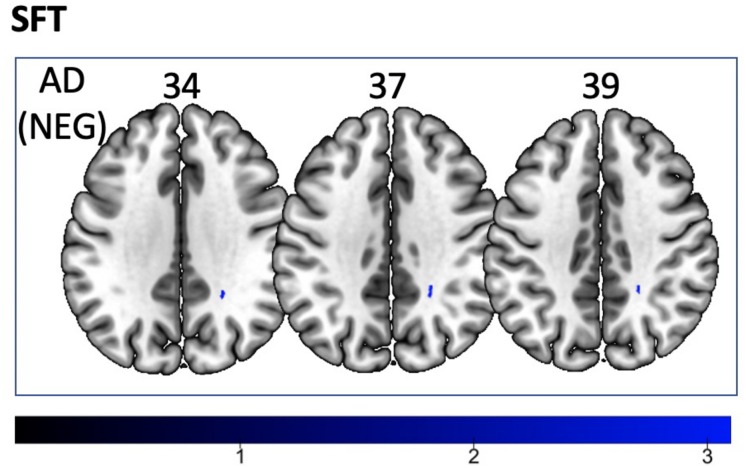
Significant correlations between SFT scores and AD values (*p* < 0.05, cluster-wise FDR corrected for multiple comparisons). Pictures are in neurological convention with left side in the figure corresponding to the left side of the brain.

**FIGURE 4:**
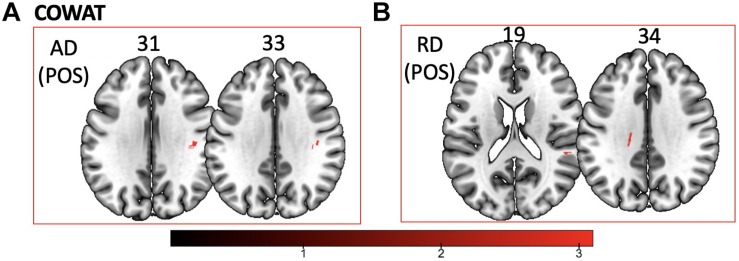
Significant correlations between COWAT scores and **(A)** AD and **(B)** RD values (*p* < 0.05, cluster-wise FDR corrected for multiple comparisons). Pictures are in neurological convention with left side in the figure corresponding to the left side of the brain.

**FIGURE 5:**
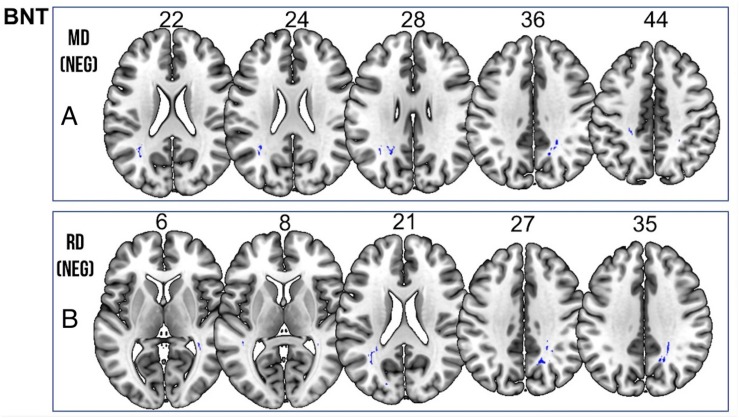
Significant correlations between BNT scores and **(A)** MD and **(B)** RD values (*p* < 0.05, cluster-wise FDR corrected for multiple comparisons). Pictures are in neurological convention with left side in the figure corresponding to the left side of the brain.

We found a *positive* correlation between increased FA and language performance on PPVT, SFT, and BNT within several white matter regions discussed below (Figures 2A,B). There was a *positive* correlation between increased SFT and PPVT and increased FA within the posterior portion of the right inferior fronto-occipital fasciculus (Figure 2A). In contrast, there was a *negative* correlation between AD and SFT scores within the posterior right inferior fronto-occipital fasciculus (Figure 3). For COWAT, there was a *negative* association between increased FA within the body of the corpus callosum on the left (Figure 2A) associated with decreased COWAT scores. Conversely, there was *positive* correlation between AD and COWAT scores within the right SLF (Figure 4A) and between RD and COWAT scores in the body of the corpus callosum on the left (Figure 4B). For the BNT, there was a *positive* correlation between BNT scores and FA within the left SLF, and in the posterior and anterio-lateral portions of the right inferior fronto-occipital fasciculus (Figure 2A). There was a *negative* correlation between MD (Figure 5A) and RD (Figure 5B) and BNT scores within both the left SLF, and the right inferior fronto-occipital fasciculus. Finally, there were no significant correlations between DTI metrics and CI-BDAE. The MNI values for the results of each regression analysis between each of the language tests and DTI metrics are provided in Tables 2–5.

**TABLE 2:** Locations of regions showing significant relationships between FA and behavioral scores indicated in Figure 2A.

**Language outcome**	**#voxels in cluster**	***t*-value (peak)**	**MNI coordinates (*x y z*)**		
	105	4.862	−17	−24	34
COWAT		3.632	−19	−23	39
		3.513	−14	−16	32
		3.342	−14	−22	31
		3.275	−18	−18	37
	84	4.923	−35	−54	23
		4.797	−33	−52	21
		4.754	−36	−53	22
		3.984	−32	−62	25
		3.901	−34	−56	22
	89	4.478	16	−58	37
		4.011	19	−56	39
BNT		3.834	21	−55	43
		3.528	19	−58	42
		3.352	21	−61	37
	49	4.17	36	−42	6
		3.911	37	−43	5
		3.872	35	−41	7
		3.582	37	−49	4
		3.579	37	−47	4
	108	4.399	37	−52	1
PPVT		4.047	35	−42	7
		4.022	37	−44	5
		3.981	42	−47	−1
		3.979	40	−50	1
	77	4.507	37	−34	4
SFT		4.295	35	−39	8
		4.076	37	−38	2
		3.94	37	−37	6
		3.857	38	−31	4

*All local maximal FA values separated by more than 1 mm; *x*, *y*, and *z* are Montreal Neurological Institute (MNI) coordinates in the left–right, anterior–posterior, and inferior–superior dimensions, respectively.*

**TABLE 3:** Locations of regions showing significant negative relationships between AD and performance on the SFT as shown in Figure 3.

**Language outcome and diffusion metric**	**Extent**	***t*-value**	**MNI coordinates (*x y z*)**		
SFT and AD	66	4.633	24	−49	34
	66	4.600	22	−45	39
	66	3.427	21	−49	38

*All local maximal AD values separated by more than 1 mm; *x*, *y*, and *z* are Montreal Neurological Institute (MNI) coordinates in the left–right, anterior–posterior, and inferior–superior dimensions, respectively.*

**TABLE 4:** Locations of regions showing significant positive relationships between COWAT performance and AD (shown in Figure 4A) and RD (shown in Figure 4B).

**Language outcome and diffusion metric**	**Extent**	***t*-value**	**MNI coordinates (x y z)**		
COWAT and AD	81	4.843	43	−20	31
	81	4.792	42	−21	34
	81	4.539	40	−25	32
	81	3.870	40	−23	31
	81	3.790	37	−25	33
	66	5.362	23	−45	37
	66	4.932	24	−47	34
COWAT and RD	70	4.517	−18	−24	34
	70	4.275	−17	−25	32
	70	4.026	−14	−17	32
	50	4.393	52	−37	19
	50	3.967	41	−39	22
	50	3.873	54	−37	19
	50	3.651	45	−40	25

*All local maxima AD and RD values separated by more than 1 mm; *x*, *y*, and *z* are Montreal Neurological Institute (MNI) coordinates in the left–right, anterior–posterior, and inferior–superior dimensions, respectively.*

**TABLE 5:** Locations of regions showing significant negative relationships between BNT performance and MD (shown in Figure 5A) and RD (shown in Figure 5B).

**Language outcome and diffusion metric**	**Extent**	***t*-value**	**MNI coordinates (*x y z*)**		
BNT and MD	39	5.002	27	−48	38
	39	3.774	29	−49	37
	39	3.698	26	−47	42
	93	4.891	24	−46	34
	93	4.344	25	−48	32
	93	4.314	23	−52	32
	93	3.769	21	−53	36
	143	4.386	−33	−51	25
	143	4.136	−34	−52	23
	143	4.127	−33	−54	24
	143	4.07	−33	−58	22
	30	4.383	−23	−36	44
	30	3.881	−22	−36	42
	30	3.833	−25	−34	44
	30	3.658	−22	−36	46
	31	4.025	18	−53	36
	31	3.736	17	−58	36
	31	3.683	16	−56	35
	62	3.98	−22	−58	28
	62	3.852	−21	−53	30
	62	3.741	−23	−55	28
	62	3.535	−24	−51	30
BNT and RD	33	4.650	27	−48	38
	33	3.597	28	−45	38
	261	4.599	24	−44	35
	261	4.551	19	−58	36
	261	4.278	25	−41	35
	250	4.525	−33	−59	23
	250	4.495	−35	−54	23
	250	4.475	−33	−53	22
	250	4.434	−34	−57	23
	64	4.306	−40	−46	17
	64	3.761	−42	−44	11
	64	3.743	−42	−39	8
	64	3.415	−42	−42	10
	79	4.130	−22	−58	28
	79	4.067	−27	−48	29
	79	3.883	−26	−51	28
	79	3.797	−23	−55	27
	40	4.095	36	−41	6
	40	3.864	37	−47	4
	40	3.835	37	−45	4
	40	3.687	37	−49	4
	40	3.447	35	−43	9
	40	3.853	−17	−79	15
	40	3.820	−18	−81	17
	40	3.647	−18	−82	21
	36	3.809	−23	−36	44
	36	3.698	−22	−38	47
	36	3.580	−21	−42	46
	36	3.557	−22	−40	45

*All local maximal MD and RD values separated by more than 1 mm; *x*, *y*, and *z* are Montreal Neurological Institute (MNI) coordinates in the left–right, anterior–posterior, and inferior–superior dimensions, respectively.*

### Age Related Differences in White Matter DTI Metrics

**FIGURE 6:**
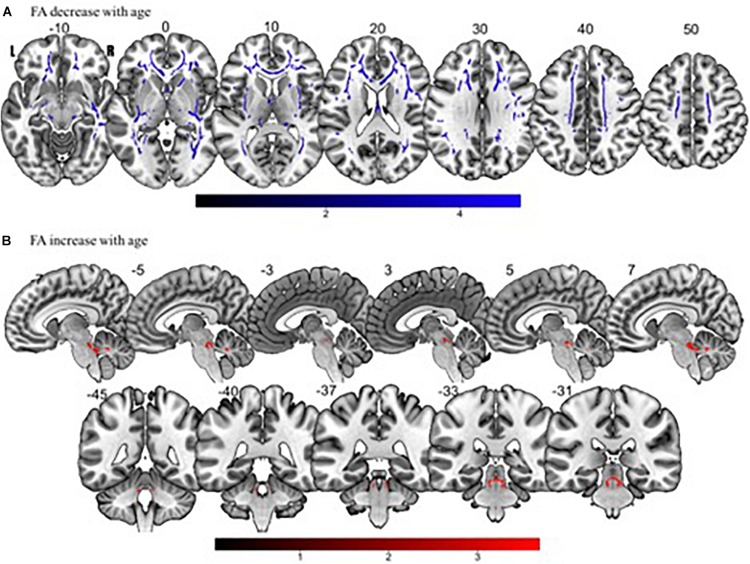
Significant correlations between **(A)** FA decrease with age and **(B)** FA increase with age (*p* < 0.05, cluster-wise FDR corrected for multiple comparisons). Pictures are in neurological convention with left side in the figure corresponding to the left side of the brain.

**FIGURE 7:**
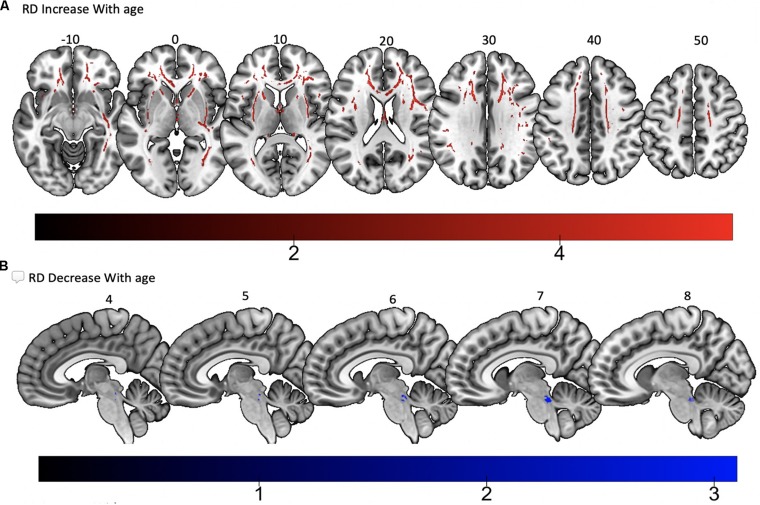
Significant correlations between **(A)** RD increase with age and **(B)** RD decrease with age (*p* < 0.05, cluster-wise FDR corrected for multiple comparisons). Pictures are in neurological convention with left side in the figure corresponding to the left side of the brain.

**FIGURE 8:**
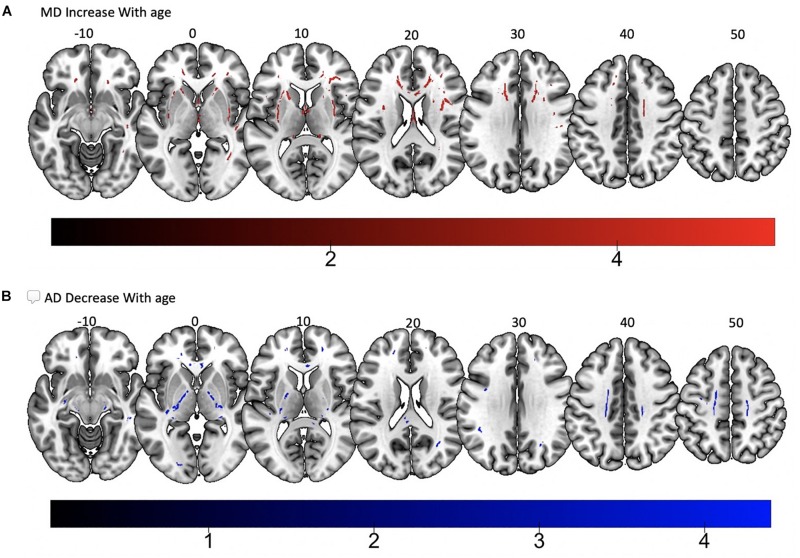
Significant correlations between **(A)** MD increase with age and **(B)** AD decrease with age (*p* < 0.05, cluster-wise FDR corrected for multiple comparisons). Pictures are in neurological convention with left side in the figure corresponding to the left side of the brain.

Tract-based spatial statistics regression analyses were performed on all subjects and computed between individual FA, AD, MD, and RD maps and the subjects’ age (controlled for sex, handedness, and MRI scanner). These analyses revealed a diffuse linear *decrease* in white matter FA with age (Figure 6A) and a corresponding global *increase* in RD (Figure 7A). Additionally, we found age related *increases* in MD which were more prominent within the anterior > posterior white matter tracts (Figure 8A) and *decreases* in AD within the central white matter structures (Figure 8B). We also found a significant focal *increase* in FA (Figure 6B) and a corresponding decrease in RD (Figure 7B) within the bilateral superior cerebellar peduncles (SCPs) with increasing age.

### Sex Differences in White Matter DTI Metrics

A two sample *t*-test was used to compare white matter FA in males vs. females using age as an interaction variable and corrected for handedness. With increasing age, FA decreased faster within the right SLF in males compared to females. However, there were no significant differences noted on further analysis of AD, RD, and MD. There was no association between handedness and any white matter DTI metric, regardless of sex.

### Scanner Effect

To assess for possible differences between data collected on different scanners, 44 subjects from the Cincinnati group were randomly selected and compared to 44 matched for age, sex, and handedness subjects from UAB. A two sample *t*-test was used to compare white matter FA, AD, RD, and MD and behavioral scores between the two scanner groups using the behavioral score as an interaction variable and corrected for handedness, sex, and age. No significant differences were found between groups in the relationship between behavior scores and DTI measures. This process was repeated five times using different Cincinnati subject pools but having matching characteristics to the UAB participants, all with the same negative result. Additionally, demographic and performance characteristics between each scanner, not including DTI metrics, are shown in Table 1. Age and sex were equally distributed across scanners. The only statistically significant difference noted was between the mean PPVT score in each scanner, however, this small numerical difference is of unclear clinical significance.

## Discussion

We show a linear correlation between increasing age and global reduction in white matter FA and a corresponding global increase in RD in a large group of participants spanning the ages between 17 and 76. These findings are consistent with prior studies that showed decreases in whole brain white matter volumes with increasing age (Liu et al., 2016, 2017). Further, prior investigations have suggested a decline in white matter volume after the age of 60, but for the purposes of this study, we did not dichotomize the age variable as <60 vs. >60 years of age. However, we did perform a polynomial regression analysis which revealed the same distribution as our linear regression model. This finding is most likely due to a low representation of subjects at the extremes of age. Physiologically, the decrease in white matter FA is thought to represent age-related alterations in myelin integrity and structure (Ziegler et al., 2010). Our study helps to corroborate these findings by showing a corresponding global increase in RD, which also suggests demyelination, as opposed to loss of axonal integrity (Song et al., 2003). Studies in aging primates have shown varying forms of myelin disruption including myelin lamellae splitting and the presence of holes in the myelin sheath (Peters, 2002). Another study compared five healthy young female brains (mean age 38) and five healthy elderly brains (mean age 74) and found that the length but not the volume of myelinated fibers was significantly reduced in the elderly population and also suggested that thin but not thick myelinated fibers were lost in elderly group (Tang et al., 1997). Unfortunately, there is a lack of human data correlating diffusion MR metrics and subsequent post-mortem histopathology in healthy adult brains. Thus, whether changes in white matter metrics result from volume loss in myelinated fibers, a decrease in the overall number of myelinated fibers, or a disruption in the integrity of fibers remains unclear.

We found a significant relationship between increasing age and better language performance on BNT and PPVT. However, these effects were minimal (both *R*^2^s = 0.02) and likely representing the fact that language improves or remains consistent until 40–60 years of age. The lack of participants at the extremes of age may have also contributed to the best fit analysis showing an increase in language performance on these tests; this finding is not inconsistent with prior literature (Albert et al., 1988). Overall, this study failed to show a significant decline in language testing scores in our aging population, despite global reduction in FA. However, we did find a correlation between increased FA and a corresponding decrease in other DTI metrics and better performance on language testing in certain tracts as discussed below.

Analyses of findings located within specific white matter tracts and language scores showed that increased FA within the non-dominant posterior portion of the IFOF correlated with higher scores on three of the four language assessments after controlling for handedness, sex, and scanner type. Alternatively, lower FA within the non-dominant IFOF was associated with poorer language testing scores. Within this region, AD, RD, and MD were found to be inversely proportional to FA, with AD showing a negative correlation with SFT, and RD and MD showing a negative correlation with BNT. Decreases in FA that correspond with increases in RD and AD suggest possible changes in either the degree of axonal or myelin degeneration. Specifically, increases in AD correspond to axonal degeneration, while increases in RD correspond to loss of myelin (Song et al., 2003). Thus, changes in axonal integrity within the IFOF may affect sematic fluency, while changes in myelination may affect word retrieval.

The IFOF is involved in semantic language processing. Intraoperative stimulation of this tract produced semantic paraphasias (Martino et al., 2010). Our data suggest that an increase in white matter FA, and corresponding decreases in MD, RD, and AD, within the non-dominant IFOF may be associated with improved semantic language function – this finding is corroborated by our previous findings of changes in fMRI signal in the non-dominant hemisphere supporting better semantic performance (Donnelly et al., 2011). Some have argued that activation in the non-dominant hemisphere is compensatory and/or counteracting neurocognitive decline with progressing age (Cabeza et al., 2002; Szaflarski et al., 2002, 2013; Nenert et al., 2017, 2018). This is evidenced by studies showing an association between maintenance or an increase in naming accuracy in older adults with bi-hemispheric fMRI participation (Wierenga et al., 2008). In contrast, others argue that non-dominant hemisphere activation is associated with an overall higher level of language functioning. In healthy adults, it has been shown that right hemisphere activation is associated with higher level language tasks (e.g., comprehending metaphors and jokes), increase in linguistic complexity of sentences, and better performance in multiple language tasks including verbal fluency, naming, and reading (Just et al., 1996; Coulson and Wu, 2005; Mashal et al., 2007; van Ettinger-Veenstra et al., 2010). Our behavioral testing analysis, while controlling for age, showed that increased FA within several language pathways of the non-dominant hemisphere was associated to better language scores. This finding does not disprove that non-dominant hemisphere support for language is compensatory with progressing age, but rather, suggests that better bilateral structural connectivity results in better overall language function, regardless of age. Additionally, our findings of increased white matter FA in the non-dominant hemisphere add further evidence to suggest that the classical model of language lateralization (Geschwind and Galaburda, 1985) is incomplete, and vastly underrepresents the interconnectivity of multiple brain regions associated with language function.

Interestingly, we found a linear increase in FA and corresponding decrease in RD, within the bilateral SCPs with increasing age. The superior and middle cerebellar peduncles play a major role in the cortico-cerebellar-cortical loop, having projections from multiple cortical areas to the contralateral cerebellar cortex. These pathways pass through the pontine nuclei and connect to different regions in the cerebral cortex through a variety of thalamic nuclei (Naidich and SpringerLink, 2009). In contrast to the inferior and middle peduncles, the SCP is the main efferent pathway of the cerebellum. It plays an important role in coordination of the ipsilateral arm and leg. Prior investigations using voxel based analysis of healthy adults described a linear association with age and white matter atrophy in the SCP (Pagani et al., 2008). Based on this prior study, one would expect to find a decrease in FA within the SCP with increasing age. One possible explanation for our finding is that white matter volume is thought to remain stable or increase slightly through adulthood, possibly peaking in the 4th decade (Pagani et al., 2008). The mean age of subjects in our population was 40 years. Additionally, while the cerebellum seems to be affected by age, with volumes declining after age 50 (Luft et al., 1999), its loss of volume is less as compared to the cerebrum. Therefore, an outflow tract (SCP) from the cerebellum may atrophy later in the aging process compared to global cerebral white matter and the inflow tracts from the cerebrum to the cerebellum (inferior and middle cerebellar peduncles). Further analyses on this particular white matter region, utilizing additional quantitative white matter analysis techniques may be required in the future to confirm this finding.

The findings from the current study should be considered in the context of its limitations. One area of concern is the fact that different MRI scanners were used for data acquisition with an imbalance in the number of participant data from one scanner. To test whether this limitation affected our results, we performed comparison analyses between 44 participants from the UAB cohort and 44 matched participants from the Cincinnati cohort. This regression was performed over five different matched groups to maximize reliability. We found no significant difference between scanners regarding behavioral testing scores and white matter FA, MD, RD, or AD, which suggests that the scanner effect on our results were minimal. Also, the relatively fewer number of participants over the age of 70 may have limited our ability to detect relationships between the DTI metrics and language performance in more elderly participants. However, overall, our study adds to our knowledge of relationships between white matter microstructure and specific language functions in healthy aging.

## Data Availability Statement

The datasets generated for this study are available on request to the corresponding author.

## Ethics Statement

This study was carried out in accordance with the recommendations of the University of Cincinnati (UC), Cincinnati Children’s Hospital Medical Center (CCHMC), and University of Alabama at Birmingham (UAB) Institutional Review Boards with written informed consent from all subjects. All subjects gave written informed consent in accordance with the Declaration of Helsinki. The protocol was approved by the University of Cincinnati (UC), Cincinnati Children’s Hospital Medical Center (CCHMC), and University of Alabama at Birmingham (UAB) Institutional Review Boards.

## Author Contributions

JS collected the funds. JH performed the literature search. JS designed the study. RN performed the statistical analysis. JH, RN, JA, AG, and JS interpreted the data. JH and JS prepared the manuscript. RN, JA, AG, JS, and JH reviewed the manuscript and approved the content.

## Conflict of Interest

The authors declare that the research was conducted in the absence of any commercial or financial relationships that could be construed as a potential conflict of interest.

## Footnotes

1 https://www.fil.ion.ucl.ac.uk/spm/
